# Intelligent modeling of hydrogen sulfide solubility in various types of single and multicomponent solvents

**DOI:** 10.1038/s41598-023-30777-8

**Published:** 2023-03-07

**Authors:** M. A. Moradkhani, S. H. Hosseini, K. Ranjbar, M. Moradi

**Affiliations:** 1 Department of Chemical Engineering, Ilam University, Ilam, 69315-516 Iran; 2 Department of Chemical Engineering, Faculty of Engineering, Shahid Chamran University of Ahvaz, Ahvaz, Iran

**Keywords:** Engineering, Chemical engineering

## Abstract

This study aims to study the solubility of acid gas, i.e., hydrogen sulfide (H_2_S) in different solvents. Three intelligent approaches, including Multilayer Perceptron (MLP), Gaussian Process Regression (GPR) and Radial Basis Function (RBF) were used to construct reliable models based on an extensive databank comprising 5148 measured samples from 54 published sources. The analyzed data cover 95 single and multicomponent solvents such as amines, ionic liquids, electrolytes, organics, etc., in broad pressure and temperature ranges. The proposed models require just three simple input variables, i.e., pressure, temperature and the equivalent molecular weight of solvent to determine the solubility. A competitive examination of the novel models implied that the GPR-based one gives the most appropriate estimations with excellent AARE, R^2^ and RRMSE values of 4.73%, 99.75% and 4.83%, respectively for the tested data. The mentioned intelligent model also performed well in describing the physical behaviors of H_2_S solubility at various operating conditions. Furthermore, analyzing the William's plot for the GPR-based model affirmed the high reliability of the analyzed databank, as the outlying data points comprise just 2.04% of entire data. In contrast to the literature models, the newly presented approaches proved to be applicable for different types of single and multicomponent H_2_S absorbers with AAREs less than 7%. Eventually, a sensitivity analysis based on the GPR model reflected the fact that the solvent equivalent molecular weight is the most influential factor in controlling H_2_S solubility.

## Introduction

Hydrogen sulfide (H_2_S), as the most common acid gases, is produced in several industries, such as wastewater treatment, coal synthesis, and oil and gas production^1–8^. In almost all these industries, H_2_S removal is a vital step due to its high corrosion, resulting in equipment and pipeline damage^9–11^. Moreover, H_2_S is one of the major sources of air pollution and acid rain^12^. On the other hand, the high toxicity of H_2_S poses serious health risks to humans, plants, and other creatures^13–16^. Accordingly, various technologies have been developed for H_2_S removal from gas mixtures^17–21^. Among them, chemical absorption is broadly used in various units as it is inexpensive and highly flexible^22^. Since the solubility of H_2_S in various types of solvents is critical in industrial simulation and design, it is imperative to develop comprehensive and exact predictive models applicable for a wide range of solvents and operating conditions.

Several experimental investigations are available in the literature on the H_2_S solubility in various types of solvents. The most commonly used solvents involved in numerous industries are aqueous solutions of alkanolamines^23–33^. These solvents can be divided into three main categories, namely, primary, secondary and ternary amines. While alkanolamines show high capabilities during H_2_S removal process, their disadvantages such as, amine loss during regeneration, corrosion caused by degradation, water transfer into gas steam, and high cost of the process restrict their applications in some industrial units^34–37^. Accordingly, various alternative solvents have been developed to resolve such disadvantages. The ionic liquids can be considered as the most capable solvents because of their high stability, recyclability, flexibility, and also their low vapor pressure^38–45^. Moreover, they do not cause pollution, which makes them less hazardous for the environment^42,44,46–48^. The performances of several types of chemical absorbents, including organic liquids, electrolytes, etc. have also been investigated for H_2_S removal from sour gas^47,49–54^.

There are several empirical and thermodynamic-based models for estimating the H_2_S solubility in different types of solvents. However, they are mostly applicable for special conditions^5,26,55–62^. Also, these models are much more complex to calculate solubility, which limits their usage. Haghtalab and Mazloumi^63^ utilized electrolyte cubic square-well equation of state^64^ to predict the H_2_S solubility in aqueous solutions of MDEA. The model showed the AARE of 11.4% for 189 data points for H_2_S–H_2_O–MDEA systems. Al-Rashed and Ali^65^ developed a model for predicting the acid gases (CO_2_ and H_2_S) loading in MDEA and DEA aqueous solutions based on electrolyte–UNIQUAC method. The model provided satisfactory agreements with 2854 experimental data. Soltani Panah^66^ employed the CPA equation of state to model the H_2_S solubility in ionic liquids, and determined the pure parameters of ionic liquids based on experimental data for density and vapor pressure. The established model exhibited an average deviation less than 10% for all analyzed ionic liquids. Yazdi et al.^67^ suggested a model based on PC-SAFT equation of state for H_2_S-H_2_O-MDEA systems and 295 experimental data, which resulted the average relative error of 0.0001% in estimation the bubble pressure. In another study, it was shown that RETM equation of state can predict H_2_S solubility in various ionic liquids^68,69^.

Since the thermodynamic equations of state for H_2_S solubility in solvents need extra fluids information, machine learning algorithms can be chosen as alternative predictive approaches in the field^70,71^. In most of earlier studies in the field, the experimental data for solubility of H_2_S in ionic liquids have been used for developing the models^35,46,72–74^. An extended review in this regard was presented by Yusuf et al.^75^. Ahmadi et al.^76^ utilized genetic programming approach to develop an explicit correlation for H_2_S solubility in 11 different ionic liquids. The correlation showed a total AARE of 4.38% for all 465 analyzed data. Amedi et al.^77^ evaluated different types of machine learning algorithms to model the H_2_S solubility in ionic liquids based on 664 experimental data. Among them, the MLP approach provided the most reliable predictions with AARE and R^2^ values of 11.68% and 99.51%, respectively for test data. In another related work, the extreme learning machine (ELM) approach was employed by Zhao et al.^37^ to predict H_2_S solubility in ionic liquids. This model was established based on 1282 experimental data for 27 ionic liquids, utilizing pressure, temperature and number of fragments as input factors. It should be noted that the AARE of 5.78% was obtained by ELM-based model during test stage. Barati-Harooni et al.^78^ proposed various intelligent methods to approximate H_2_S absorption in in 14 ionic liquids. Beside the temperature, pressure and molecular weight of ionic liquids, 9 structural-related factors were also defined as models' inputs. It was found that the least square support vector machine (LSSVM) has the superior predictions with total AARE of 0.13% for 664 experimental data. A similar observation about the ability of LSSVM in the field was reported by Baghban et al.^36^. Kang et al.^79^ utilized the ELM methods to estimate the H_2_S mole fraction in 28 different ionic liquids, considering the electrostatic potential surface of molecules as one of the model inputs. The analyzed database included 1318 experimental data, and the ELM method showed an AARE of 5.07% for the tested data. Amar et al.^80^ provided a competitive evaluation of machine learning algorithms to model the H_2_S solubility based on 1243 data points for 33 ionic liquids. The models' inputs were pressure, temperature, acentric factor, critical pressure and critical temperatures of ionic liquids. It was found that the results of advanced committed machine intelligent system (CMIS) are much better than those of the conventional methods such as multilayer perceptron. More recently, Mousavi et al.^81^ examined the application of deep learning algorithms for estimating the H_2_S solubility in ionic liquids based on 1516 data points, and considered the chemical structural of molecules as an adjusted parameter. All deep learning-based methods had satisfactory results with AAREs between 3.20% and 7.15% for test data.

From the above literature survey, it is evident that the earlier models developed for H_2_S solubility have been validated with data for limited types of solvents, particularly ionic liquids. Also, none of them are well verified for multi-component solvents. On the other hand, the application of machine learning algorithms to design universal models applicable for various operating conditions and H_2_S absorbers has not been investigated so far. Therefore, to address the above deficiencies, in the present communication, an immense set of experimental data, including 5148 samples is gathered from 54 published sources, which is the widest H_2_S solubility databank analyzed to date. The solubility data for 95 single and multicomponent solvents such as amines, ionic liquids, electrolytes, organics, etc. are covered by the current data. To build robust models, three well-known intelligent approaches of MLP, GPR and RBF are used, among which the GPR approach is used in practice for the first time. In order to clarify the predictive ability of the newly established models, their accuracy for different types of solvents, both single and multi-component solvents, is evaluated using statistical criteria. The reliability of the experimental data used to design the new model is also examined through the William's plot. Furthermore, the influences of operating conditions on H_2_S solubility are studied using the models' outcomes, then the most effective factors are introduced. A comparative assessment between the performances of the novel intelligent models and those proposed in the earlier studies is also carried out.

## Materials and methods

### Machine learning algorithms

In this study, three well-known intelligent schemes, namely, MLP, GPR and RBF were used to design predictive models for H_2_S solubility for various single and multicomponent solvents. According to our previous studies^82–85^, these approaches have high capabilities for accurate modeling of engineering systems with nonlinear and complicated behaviors.

#### RBF

**Figure 1:**
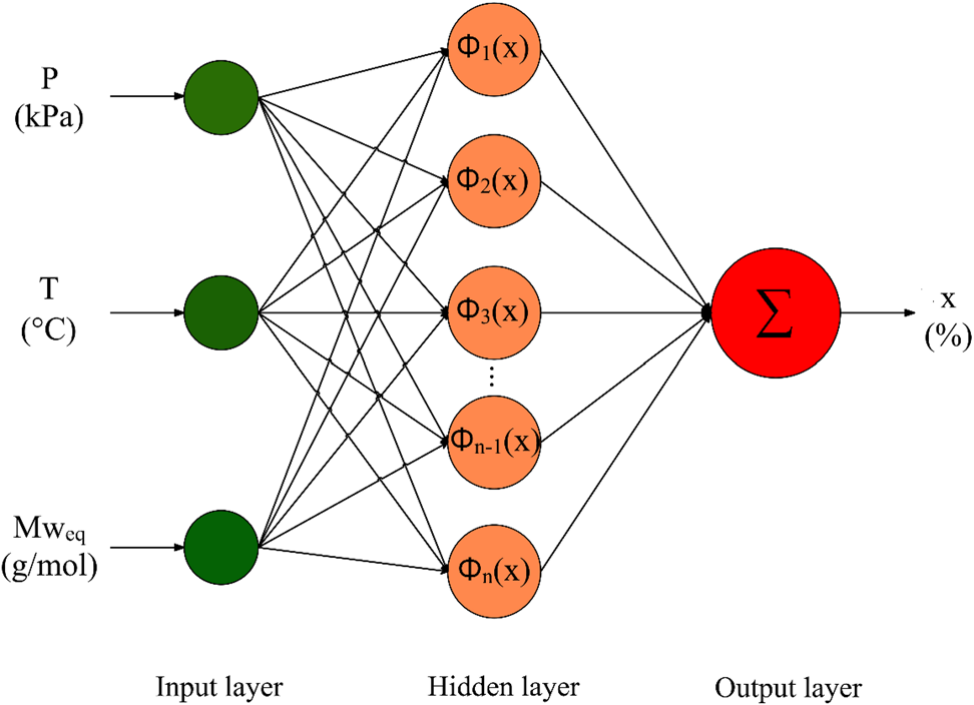
The structure of the RBF network employed for modeling of H_2_S solubility (created by Grapholite 4.0.1^90^).

The unique advantages of RBF networks, such as quick training process, uncomplicated structure and high precision modeling had made them widely used in various engineering fields. This network contains three independent layers, namely, input layer, a single hidden layer and output layer. A schematic of the RBF network employed to model the H_2_S solubility is shown in Fig. 1. As seen, the input variables are introduced to the network via the input layer. The second layer, i.e., hidden layer includes various neurons, the number of which is equal to the number of data used for training the network. These neurons have the ability to use a variety of activation functions such as Gaussian, multi-quadric, and cubic radius, etc. These functions contribute to build complex mappings between network inputs and outputs. In other words, they enable the model to adjust to the complex and nonlinear system characteristics. The Gaussian function, Eq. (1), was chosen for this study as it offered the best fit between the experimental and predicted values among a variety of activation functions. Such a result was observed in our earlier works for different systems^82,83,85–88^,

1 $${\Phi }_{i}=-\frac{{d}^{2}}{{e}^{2{\sigma }^{2}}}$$

where $\sigma$ denotes the gaussian function's standard deviation, and $d$ is the Euclidean distance between the input data and the center of network. Ultimately, the weighted sum of activation functions is presented by the output layer,

2 $$y=\sum_{k=1}^{n}{w}_{k}{\Phi }_{k}$$

It should be noted that ${w}_{k}$ is the weight of *k*th neuron in the hidden layer^89^.

#### GPR

The gaussian process regression (GPR) is known as a non-parametric and supervised machine learning algorithm which uses the concept of probability^91,92^. It should be noted that this method provides reasonable outcomes even for limited numbers of data samples. Accordingly, it is utilized for a broad range of problems with nonlinear behaviors. GPR includes a collection of random variables which they have the multivariate normal distributions^93–95^. If the outputs of $h(x)$ are estimated as $i\left(x\right)={\left[h\left({x}^{1}\right),h\left({x}^{2}\right),\dots ,h({x}^{N})\right]}^{T}$, the gaussian distribution of $h(x)$ consists of a mean function $Q={\left[Q\left({x}^{1}\right),Q\left({x}^{2}\right),\dots ,Q({x}^{N})\right]}^{T}$ and a covariance matrix $P(X,X)$ with entries ${p}_{ij}=P\left({x}^{i},{x}^{j}\right)$. Accordingly, there is a multivariate gaussian distribution for $h(x)$. It should be noted that, $P\left({x}^{i},{x}^{j}\right)$ represents the prior probability distribution of $h(x)$. Once a new set of input data to be applied, the GPR probability model is updated and a posterior probability function for $h(x)$ is calculated.

#### MLP

**Figure 2:**
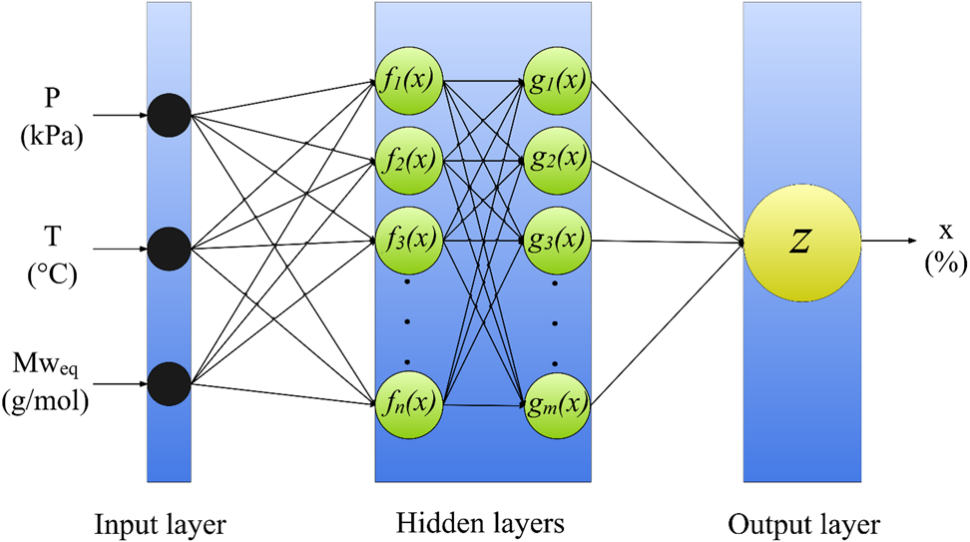
The structure of the MLP network employed for modeling of H_2_S solubility (created by Grapholite 4.0.1^90^).

MLP networks are the most common types of feed-forward neural networks, which have been designed based on nervous system of humans^96^. The main applications of these networks include pattern recognition, classification and estimation^97,98^. A schematic diagram of the MLP network used for modeling of H_2_S solubility is presented in Fig. 2. As seen, the network includes an input layer corresponding to input factors ($T$, $P$ and ${Mw}_{eq}$), another layer associated with output factors (${x}_{{H}_{2}S}$), and one or more layers between them as hidden layer(s). Each of these layers has a number of neurons, which are directly connected to the neurons in the next layer through biases and weights. The numbers of neurons in input and output layers equal to the numbers of input and output variables, respectively. However, the number of hidden layers and their corresponding neurons are adjustable. Since the MLP network is usually used for modeling of nonlinear systems, various activation functions, such as log-sigmoid, threshold and tan-sigmoid may be included in the neurons of hidden layers in order to introduce the nonlinearity to the established network. For measuring the deviation of model's outcomes from the actual data, the MLP network uses the cost function defined as follow,

3 $$\Psi =\frac{1}{2}{\left({x}_{{H}_{2}S, pre}-{x}_{{H}_{2}S, exp}\right)}^{2}$$

The back-propagation algorithm propagates the value of cost function values via the network, then the synaptic weights are accordingly tuned to minimize this value. Several training methods such as gradient descent (GD), Levenberg–Marquardt (LM) and Bayesian regularization (BR) could be employed in the back-propagation algorithm. In this study, a number of MLP network topologies were tested in order to attain the desired values of error metrics, such as AARE, RRMSE and *R*^2^. Finally, a network containing five hidden layers with [30–25–20–15–10] neurons' structure yielded the best predictions for the H_2_S solubility in solvents. In addition, the capable method of Bayesian regularization was utilized to minimize the cost function. More details about the configuration details of MLP network utilized for modeling of H_2_S solubility are summarized in Table 1.

**Table 1:** Configuration details of the MLP network utilized for modeling of H_2_S solubility.

Parameter	Type/value
Number of neurons in the input layer	3
Number of neurons in the output layer	1
Number of hidden layers	5
Neurons' structure in hidden layer	[30–25–20–15–10]
Number of weights	1800
Number of biases	101
Total number of parameters	1901
Learning role	Bayesian regularization
Train function	Trainbr
Transfer function	Tansig

It should be noted that prior to estimating, the hyperparameters of an algorithm that define the construction of a data-driven model, such as those reported in Table 1, need to be optimized. Therefore, hyperparameters are randomly tested at different values in order to determine which ones produce accurate predictions or reduce loss functions.

### Experimental data collection

In the current study, an immense experimental databank of H_2_S solubility, containing 5148 samples were collected from 54 independent sources. The operating ranges of analyzed sources are presented in Table 2. As seen, the current database covers 95 single and multicomponent solvents including amines, ionic liquids, electrolytes, organics, etc. over broad ranges of operating conditions. Therefore, it can properly satisfy the need for comprehensive experimental data in order to development of robust models.

**Table 2:** Operating ranges of analyzed sources for H_2_S solubility in solvents.

Reference	Solvent	Temperature, $(^\circ{\rm C} )$	Pressure, $({\text{kPa}})$	${x}_{{H}_{2}S}$, (%)	Number of data
^99^	MEA–sulfolane	30–100	9.3–1390.6	3.4–52	35
^100^	DEA–NMP, MEA–NMP	25–100	20.1–1301.3	2.7–65.3	43
^101^	DEA–sulfolane	30–100	14.3–1439.7	2.1–53.2	56
^102^	Dodecane	40–161	524–5675	6.69–90.2	33
^103^	Methanol, benzene	25–50	61–1180.5	1.52–48.79	41
^26^	Piperazine–water, piperazine–AMP–water, MDEA–AMP–water, MDEA–piperazine–AMP–water	40–70	202–2047	1.58–12.08	142
^104^	MDEA–water	10–15	1.06–12.72	1.05–5.54	20
^50^	DIPA–water–sulfolane	40–100	4.6–3862.3	1.57–48.77	25
^105^	[emim][EtSO_4_]	30–80	113.7–1270.4	1.2–11.8	36
^41^	[C_2_mim][eFAP]	30–80	58.2–1941.5	2.2–59.26	79
^106^	MDEA–water	40–80	1.09–313	0.98–7.49	24
^107^	2-Piperidineethanol–water	40–100	0.25–5550	1.66–38.21	37
^108^	DEA–water	37.8–148.9	0.98–3820.9	1.03–7.87	74
^109^	Methanol	25–175	63.86–8974	1.15–99.38	47
^110^	MEA, MDEA, MEA–MDEA,	40–100	0.96–445.7	0.98–9.16	164
^111^	AMP, AMP–MEA	40–100	0.53–181.6	1.01–8.47	141
^112^	MDEA–sulfolane–water	40–100	4.22–3210	0.98–10.41	32
^113^	DGA–water	50–100	2.52–1890	1.15–18.23	40
^114^	[C_2_mim][OTf]	30–80	64.3–2455.3	2.91–56.72	36
^39^	[hmim][PF_6_], [hmim][PF_4_], [hmim][Tf_2_N]	30–70	97.4–1100	2.9–53.3	97
^115^	MDEA–DEA–AMP–water, MDEA–DEA–water	40–120	2.5–1036.8	1–13.4	73
^28^	MIPA–water	40–120	51.4–1467.6	1–15.9	69
^116^	AMP–sulfolane–water	40–100	7.09–2200	0.98–3.13	18
^117^	[C_8_mim][PF_6_]	30–80	84.5–1958.4	4.63–69.72	48
^38^	[emim][PF_6_], [emim][Tf_2_N]	30–90	107.7–1933	3.2–60.9	82
^3^	[HOemim][PF_6_], [HOemim][OTF]	30–80	105.9–1839	3.62–57.95	129
^34^	[bmim][MeSO_4_]	25	10.8–750.9	2.2–52.1	8
^29^	MDEA–water	40–70	11–1065	1.76–11.42	27
^26^	MDEA–AMP–water	40–80	18.5–1441.5	4.46–11.03	31
^31^	MDEA–piperazine–water	40–120	1.26–5472.32	1.16–12.27	64
^118^	CH_3_COOH–water	40–120	1010–9708	1.12–6.68	77
^53^	NaNO_3_–water, NH_4_NO_3_–water, NaOH–water	40–120	15.1–9393	1.08–7.21	146
^119^	Na_2_SO_4_–water, (NH_4_)_2_SO_4_–water, NaCl–water, NH_4_Cl–water	40–120	1260–9784	0.99–5.57	139
^120^	Piperazine–water, MDEA–Piperazine–water	40–120	136.3–8748	2.02–13.64	103
^121^	Isooctane, N-decane, N-tridecane, N-hexadecane, squalane	50–250	192–1658	1.81–41.27	117
^52^	Propylene carbonate, dimethyl carbonate, diethyl carbonate, diethyl succinate	25–55	23–1013	1.09–54.96	465
^47^	Propylene carbonate, [Bmim][BF_4_], [Hmim][BF_4_], [Omim][BF_4_], [Omim][BF_4_]-PC	30–60	27–1030	1.27–57.35	557
^33^	MDEA–water, MDEA–AEEA–water	40–85	6.9–1398.8	1.11–9.51	131
^23^	MDEA–H_2_SO_4_–water	40–120	32.7–3866	1.01–6.58	45
^122^	DGA–water, MDEA–water	40–115	11.1–1762.64	1.05–18.45	77
^123^	DEA–water, MDEA–water, MDEA–DEA–water	40–100	7.97–1337.60	0.99–11.62	79
^124^	MDEA–water	40–120	147.9–2763	1.90–15.24	26
^125^	MDEA–water	40–140	165.2–4895.9	1.65–9.85	71
^49^	Hexane, cyclohexane, benzene	50–150	400–11,210	2.3–93.6	81
^126^	DIPA–water, DIPA–piperazine–water	40–80	19–1554	2.84–9.31	74
^127^	Propylene carbonate, sulfolane, N-methyl pyrrolidone	25–100	55.2–1654.6	2.67–70.57	66
^128^	TDG	30–50	90–1040	2.7–18.76	18
^25^	GBL, NMI	30–80	162–1356	5.7–37.5	73
^129^	NMP–water	30–80	68–1478	1–31.42	280
^130^	MDEA–piperazine–sulfolane–water, MDEA–piperazine–water, MDEA–sulfolane–water	30–80	10.3–2064.3	3.23–11.45	317
^131^	[Bzmim][Tf_2_N]	30–70	52.9–1596.2	2.47–65.12	53
^44^	[2-HEA][Ace], [B-2-HEA][Ace]	25–45	100.9–102.37	6.8–12.2	10
^132^	[emim][Ace], [emim][Pro], [emim][Lac], [bmim][Ace]	20–60	1.73–341.73	7.3–63.75	238
^68^	[DMEAH][Ac], [DMEAH][For], [MDMEAH][Ac], [MDMEAH][For]	30–60	4.62–139.51	1.03–20.91	154
Total	95 different solvents	10–250	0.25–11,210	0.98–99.38	5148

### Models' input factors

According to experimental investigations, the solubility of H_2_S in solvents mainly depends on pressure, temperature and solvents characterizations. Although the critical temperature, critical pressure, and acentric factor of solvents have been extensively utilized to assess the influence of absorber type on H_2_S solubility, these factors have not been experimentally determined for some of the solvents analyzed in this study. Additionally, using of numerical methods, such as group contribution approaches requires complicated calculations, particularly when a multicomponent absorber is used. On the other hand, some researchers have used the chemical structure of solvents to discriminate between different absorbers. This methodology extremely increases the number of input factors required to approximate solubility^37,81,133^. Since the current study aims to present simple models for H_2_S solubility, the equivalent molecular weight, which takes into account the molecular weights of all components as well as their mass fractions, has been used to consider the differences between various solvents,

4 $${Mw}_{eq}=\frac{1}{{\sum }_{i=1}^{m}\frac{{W}_{i}}{{Mw}_{i}}}$$

where ${W}_{i}$ and ${Mw}_{i}$ stand for the mass fraction and molecular weight of *i*th component, respectively. Also, $m$ denotes the number of the absorber components.

In fact, the value of equivalent molecular weight is a function of concentration and solution type. The capability of this factor to satisfy the influence of solvents characterizations has been proven in several studies^134,135^. It should be noted that each of H_2_S absorbers analyzed in this study has its unique ${Mw}_{eq}$ value, hence, this factor is capable to discriminate between various solvents, as well. Accordingly, the novel models have been established based on the following form,

5 $${x}_{{H}_{2}S} (\%)=f\left(T, P,{Mw}_{eq}\right)$$

### Error analysis

In order to examining the precisions of various models to predict the H_2_S solubility in solvents, the statistical factors of average absolute relative error (AARE), relative root mean squared error (RRMSE) and coefficient of determination (${R}^{2}$) were calculated^93,136–142^,

6 $$AARE (\%)=\frac{1}{n}\sum \left|{R}_{i}\right|\times 100$$

7 $$RRMSE (\%)=\frac{\sqrt{\frac{1}{n}\sum {\left({x}_{{H}_{2}S,exp}-{x}_{{H}_{2}S,pre}\right)}^{2}}}{\frac{1}{n}\sum {x}_{{H}_{2}S,exp}}\times 100$$

8 $${R}^{2} (\%)=\left(1-\frac{{\sum \left({x}_{{H}_{2}S,exp}-{x}_{{H}_{2}S,pre}\right)}^{2}}{\sum {\left({x}_{{H}_{2}S,exp}-\overline{{x }_{{H}_{2}S,exp}}\right)}^{2}}\right)\times 100$$

where $n$ is the total number of data. Moreover, the relative error, ${R}_{i}$ can be determined by the following equation,

9 $${R}_{i}=\frac{{x}_{{H}_{2}S,exp}-{x}_{{H}_{2}S,pre}}{{x}_{{H}_{2}S,exp}}$$

## Results and discussions

### Development of the novel predictive approaches

**Figure 3:**
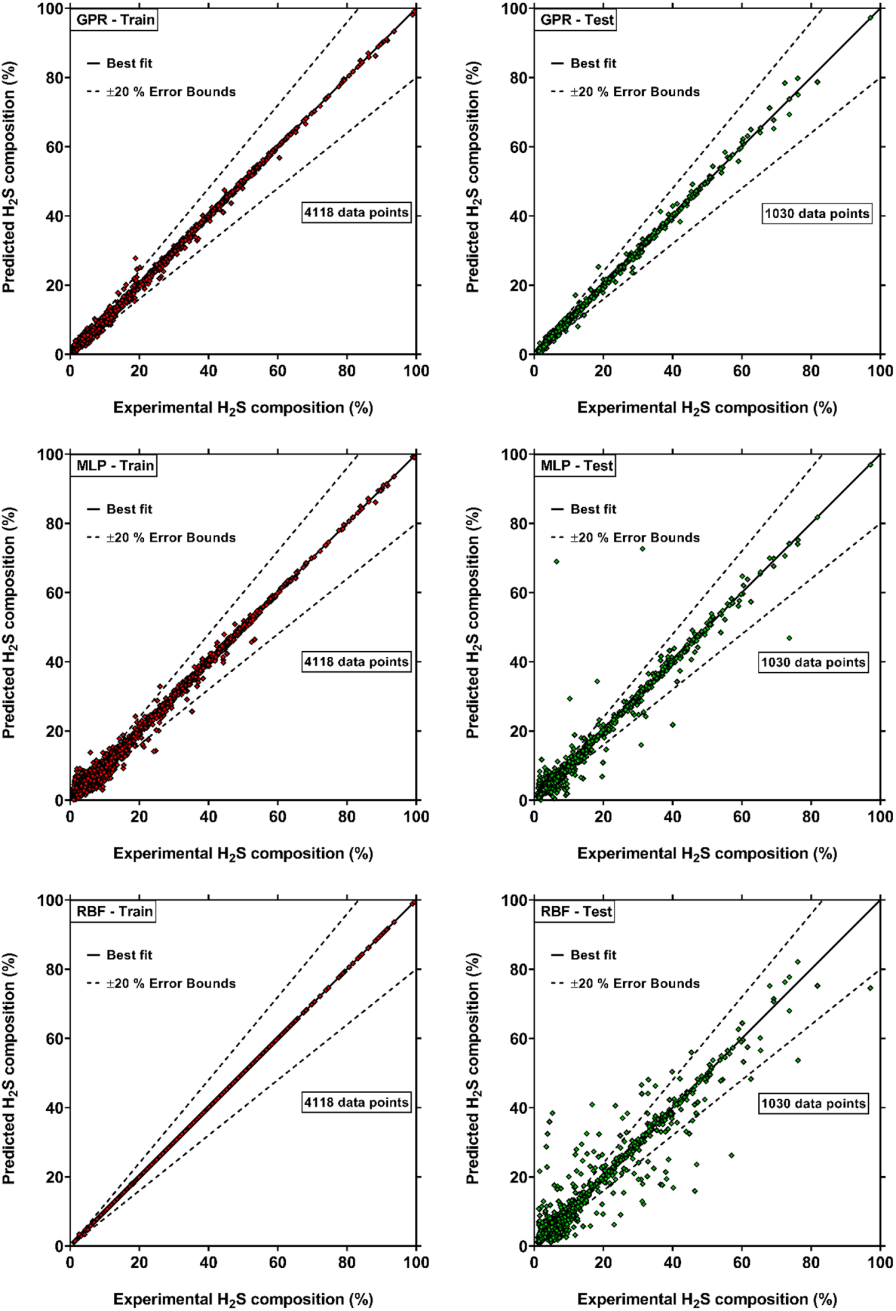
Comparison between actual values of H_2_S solubility and those predicted by the novel intelligent models (created by GraphPad 8.4.3.686^145^).

Utilizing the collected experimental data, the intelligent approaches of MLP, GPR and RBF were implemented to develop novel predictive models for H_2_S solubility based on the form presented in Eq. (2). The models were firstly trained using 4118 data points, which cover 80% of entire databank. Then, the performances of the trained models were tested by the remaining 1030 experimental data. Table 3 lists the error metrics corresponding to the novel H_2_S solubility models during training and testing steps. It is clear that the model established by the GPR approach is the only model with excellent accuracy for both train and test databases with AAREs of 4.78% and 4.73%, respectively. Moreover, its ${\mathrm{R}}^{2}$ and RRMSE values during test stage are 99.75% and 4.83%, respectively, which acknowledges its high reliability in predicting H_2_S solubility in solvents. According to classifications provided by Zendehboudi et al.^143,144^, the GPR-based model can be known as an approach with excellent prediction capability, as its RRMSE for test data is less than 10%. These results also imply the fact that the selected input variables are capable to satisfy the influences of various factors on solubility of H_2_S. The MLP-based model provides relatively good results, and has the ${\mathrm{R}}^{2}$ value of 98.41% for entire data. However, its deviations during both training and testing processes are much higher than the GPR-based model, with AARE of 16.74% and 19.66%, respectively. While the model developed by RBF approach presents the best performances during train stage, its precision for test data is unsatisfactory with AARE of 28.41%. Therefore, this model cannot be considered as a reliable predictive method. Overall, the current statistical examination shows that the GPR-based model has remarkably higher precisions in estimation of H_2_S solubility in solvents. To visualize the accuracy of the new models, their results are plotted against the actual values in Fig. 3. It is obvious that the H_2_S solubility values calculated by GPR-based approach are much closer to the best-fit line. For these reasons, it is selected as the most capable predictive model for H_2_S solubility, and the further evaluations are conducted based on this model.

**Table 3:** Error metrics of the novel intelligent models for predicting H_2_S solubility in solvents.

Error metrics	Train, (4118 data)			Test, (1030 data)			Total, (5148 data)		
	MLP	GPR	RBF	MLP	GPR	RBF	MLP	GPR	RBF
AARE (%)	16.74	4.78	0.02	19.66	4.73	28.41	17.33	4.77	5.70
${\mathrm{R}}^{2}$(%)	99.37	99.88	100	94.61	99.75	90.91	98.41	99.85	98.16
RRMSE (%)	8.21	3.61	0.14	22.46	4.83	29.17	12.88	3.92	13.84

**Figure 4:**
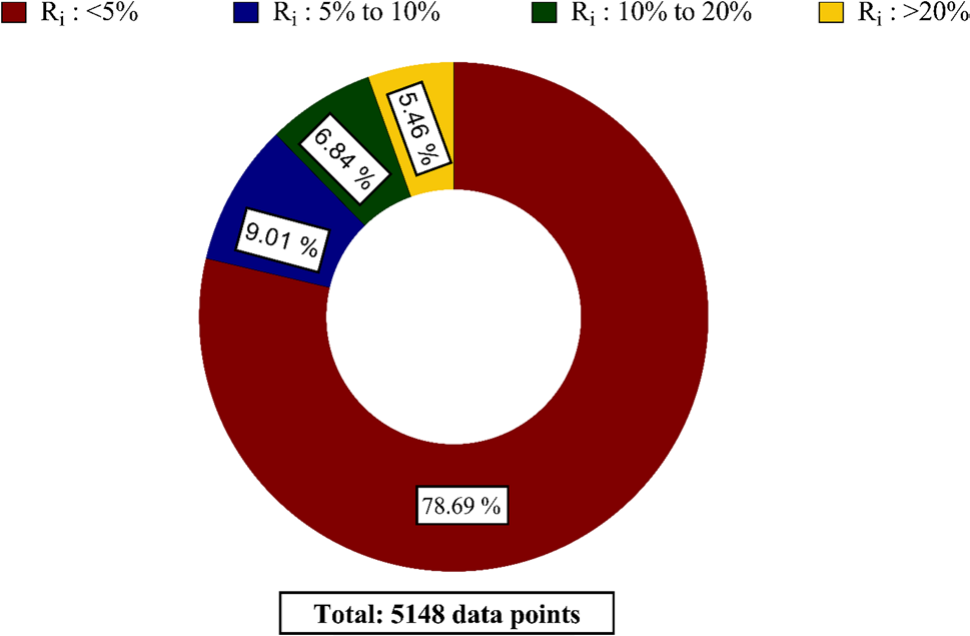
Distribution of relative error values for GPR model in various ranges (created by GraphPad 8.4.3.686^145^).

Figure 4 depicts the distribution of relative errors obtained by the GPR model in various ranges. As observed, 78.69% of H_2_S solubility values predicted by this model have relative errors less than 5% from the experimental data. Moreover, 9.01% of data fall in relative errors between 5 and 10%. So, it can be concluded that the novel model predicts more than 87% of experimental data with excellent accuracy. On the other hand, 6.84% of remaining data have also been predicted satisfactorily, as their relative errors are between 10 and 20%. This figure also reveals that just a limited number of data predicted by GPR approach (5.46% of the whole data) are beyond the ± 20% error bounds. This fact is visible from the results provided in Fig. 3. As a result, the GPR model, which has been established based on a huge number of experimental data, is an extremely high reliable predictive approach for H_2_S solubility in solvents.

### Detection of suspected data

Credibility of a predictive approach is highly dependent on precision of data employed for modeling. Suspected data are defined as those with remarkable deviations from the bulk of analyzed databank. Errors occurred during experimental measurements are the main sources of such data points. Existence of some suspected data is unavoidable when analyzing a large number experimental data from various sources^135,146–148^. In this study, the graphical technique of William's plot was employed to detect the probable suspected data. This method uses the statistical parameters of standardized residual (SR) and hat values (h_i_) to determine the mentioned data. It should be noted that the hat values are the diagonal elements of the following matrix, i.e., hat matrix,

10 $$H=X{\left({X}^{T}X\right)}^{-1}{X}^{T}$$

where $X$ is an $S\times P$ matrix, in which $S$ is the number of analyzed data samples, and $P$ indicates the number of model's input parameter. In William's plot, the data in the ranges of $-3<SR<3$ and ${h}_{i}<{H}^{*}=3(P+1)/S$ are considered as valid data points. In addition, the points with $SR>3$ or $SR<-3$ are suspected data, regardless of their hat values. Ultimately, when the conditions of $-3<SR<3$ and ${h}_{i}>{H}^{*}$ are satisfied, the corresponding data are named as ‘Good high Leverage’ points^36^. This means that while the operating conditions of these data are much deviated from the bulk of dataset, the model is capable to predicts them, precisely.

**Figure 5:**
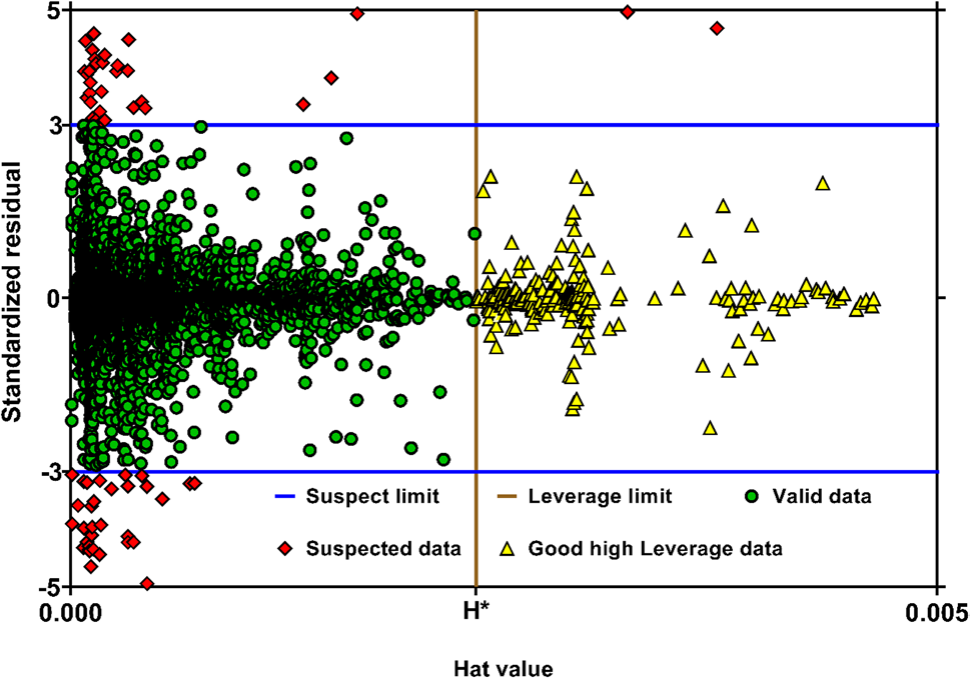
William's plot for the model proposed based on GPR method (created by GraphPad 8.4.3.686^145^).

Figure 5 demonstrates the William's plot for the model developed by GPR approach. As is clear, a huge number of points (92.87% of entire databank) are placed in the valid range. Moreover, 5.09% of data can be considered as good high Leverage points. In contrast, the suspected data cover just 2.04% of the analyzed dataset. Accordingly, it can be found that the current databank for H_2_S solubility is highly reliable, and the model proposed based on them provides exact and reasonable predictions.

### Prediction capabilities of the novel model

#### Various types of solvents

**Figure 6:**
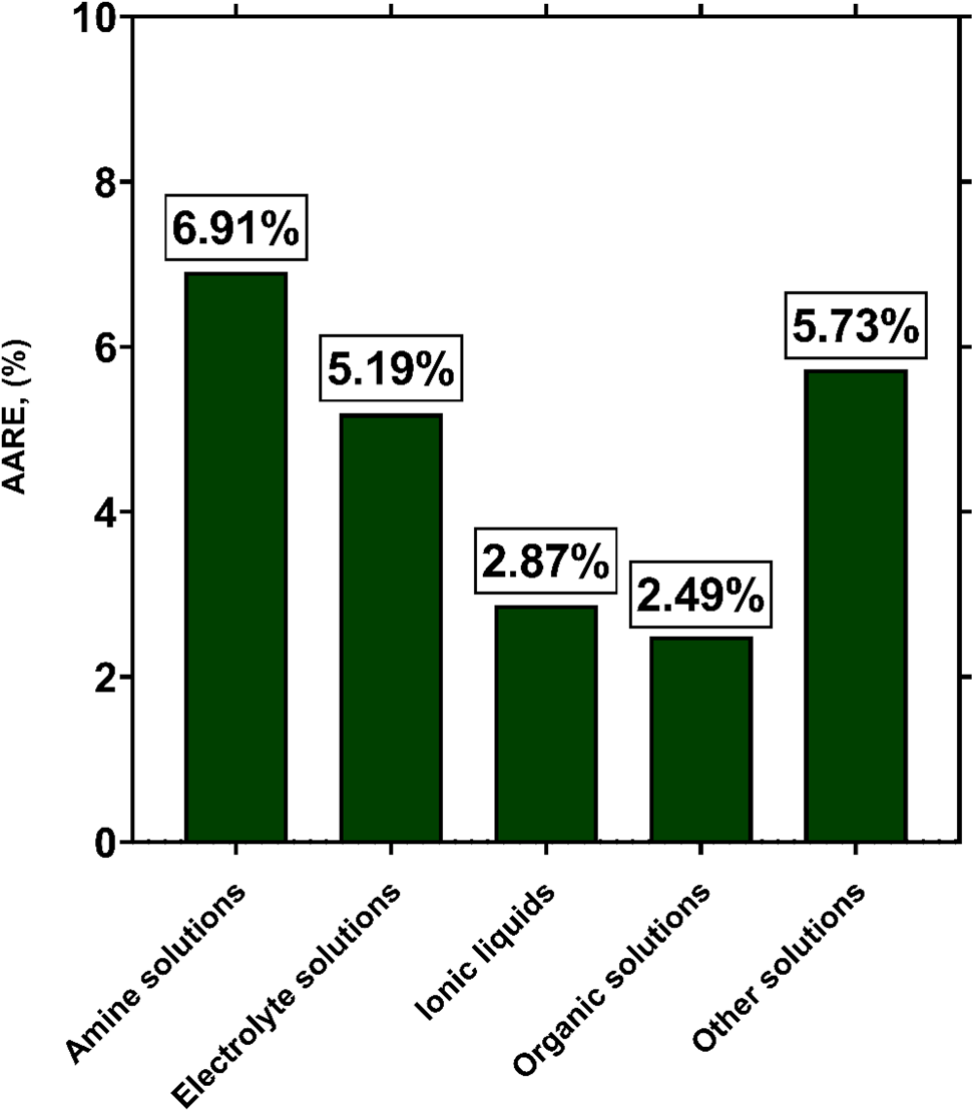
AARE values corresponding to GPR model for predicting H_2_S solubility in various types of solvents (created by GraphPad 8.4.3.686^145^).

Based on discussions provided in section “Introduction”, the earlier H_2_S solubility models have been recommended just for specific types of solvents. In contrast, the database analyzed in this study covers various types of H_2_S absorbers. Hence, the prediction capabilities of the novel model for each type of solvents should be clarified. The AARE values of GPR model for predicting H_2_S solubility in different types of solvents are exhibited in Fig. 6. As seen, the most accurate results of GPR model belong to organic solutions and ionic liquids with AAREs of 2.49% and 2.87%, respectively. In addition, it also predicts the data analyzed for H_2_S solubility in electrolyte and amine solutions with excellent AAREs of 5.19% and 6.91%, respectively, which fully acknowledges its high capability for these types of solvents. For all other types of solvents, the GPR model represents a total AARE of 5.73% from the corresponding experimental data. Overall, it can be concluded that the novel intelligent model is applicable for precise estimation of H_2_S solubility in various types of absorbers, as its AARE values in all cases do not exceed 7%.

#### Single and multicomponent solvents

**Figure 7:**
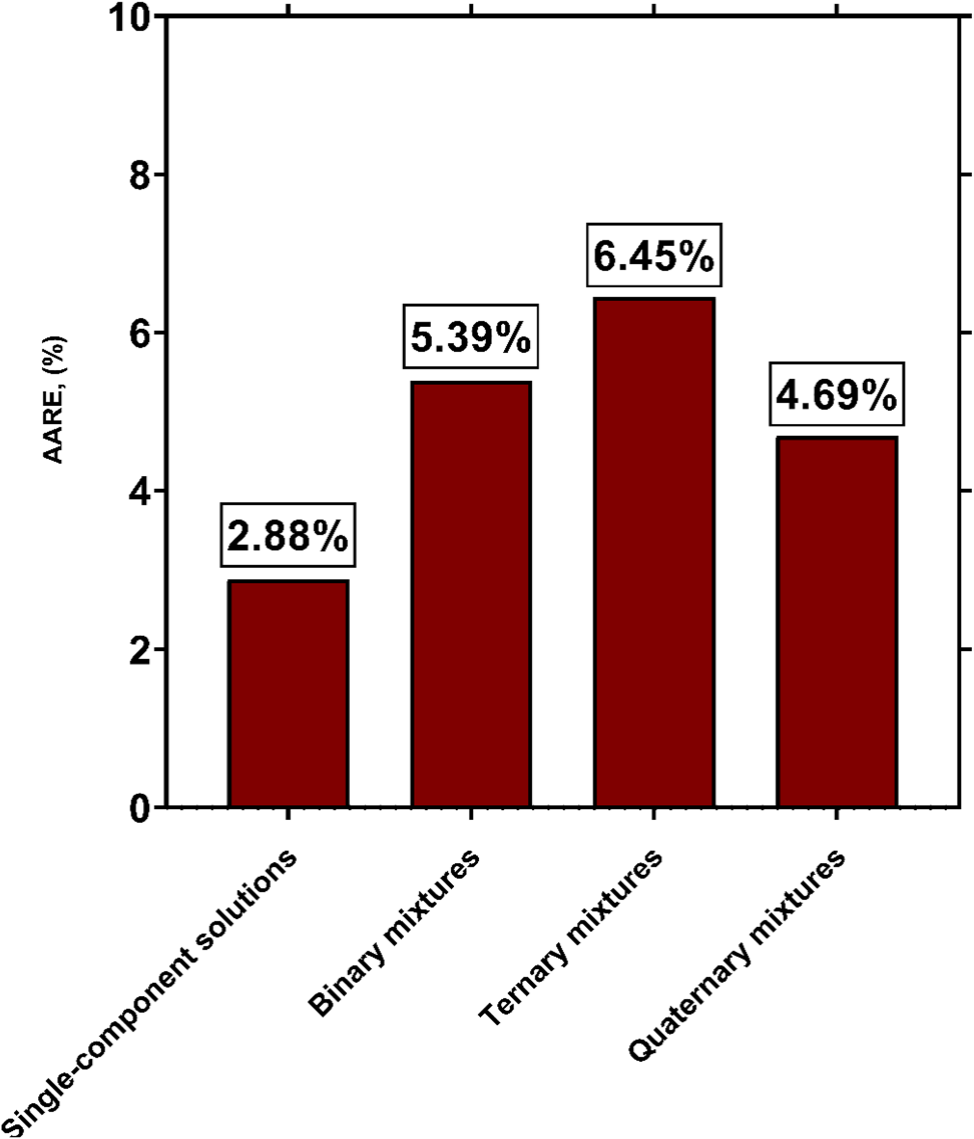
AARE values corresponding to GPR model for predicting H_2_S solubility in single and multicomponent solvents (created by GraphPad 8.4.3.686^145^).

As can be seen in Table 2, the analyzed databank includes experimental data for single-component solvents as well as those for binary, ternary and quaternary mixtures. Given the use of equivalent molecular weight as an input factor, the novel model is expected to be capable for accurate prediction of H_2_S solubility in different single and multicomponent solvents. Figure 7 illustrates the AAREs of GPR model for all analyzed cases. It is clear that the lowest deviation is related to single-component solvents with an AARE of 2.88%. Furthermore, the experimental data for binary and quaternary mixtures are also predicted, excellently, with AAREs of 4.69% and 5.39%, respectively. The highest deviation of GPR model belongs to ternary mixtures of H_2_S absorbers with AARE of 6.45%, which is highly reasonable for a predictive approach. From the current assessment, it can be found that the novel model is capable to accurately describe the H_2_S solubility in various single and multicomponent solvents. Therefore, it can be reliably utilized in scientific and engineering applications.

#### Physical trends of H_2_S solubility

In order to demonstrate the potency of the novel model for describing the physical trends of H_2_S solubility, the impacts of pressure, temperature, mass fraction of components in the solvent and the type of solvent have been studied based on the outcomes of GPR model.

**Figure 8:**
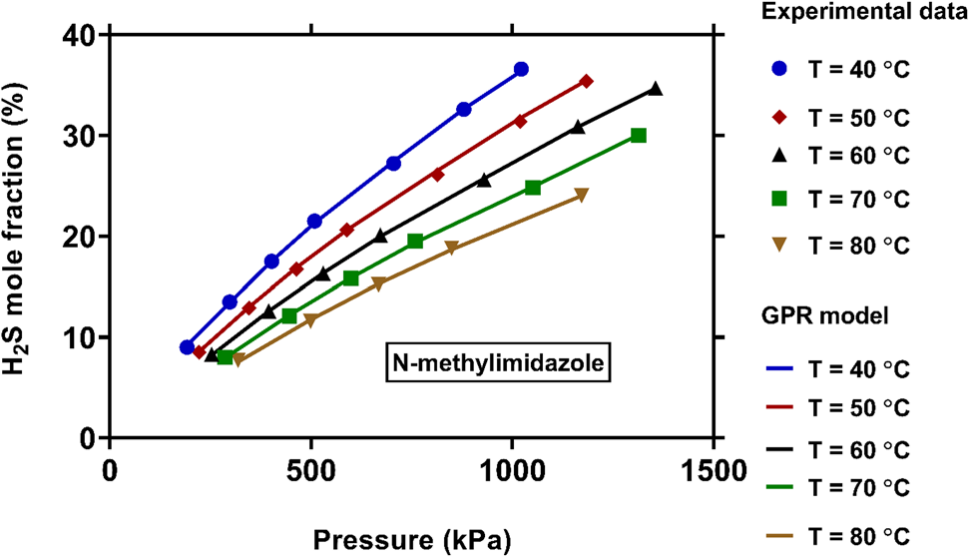
Comparison between experimental values of H_2_S solubility and those predicted by the novel model at various pressures and temperatures (created by GraphPad 8.4.3.686^145^).

Figure 8 shows the influence of pressure and temperature on solubility of H_2_S in N-methylimidazole. As expected, the H_2_S solubility is enhanced by increasing of pressure. This stems from the fact that the collision frequency and kinetic energy are increased at higher pressures, which results in higher solubility of gas. In addition, raising of temperature may reduce the H_2_S solubility in N-methylimidazole. It should be noted that a higher temperature leads to increasing the heat of system. Based on Le Chatelier's principle, the system overcomes this excess energy by inhibiting the reaction of dissolution, resulting in reduce of solubility^149^. It is clear that the novel model properly describes the impact of temperature and pressure on H_2_S solubility, and there are excellent fittings between its outcomes and experimental data.

**Figure 9:**
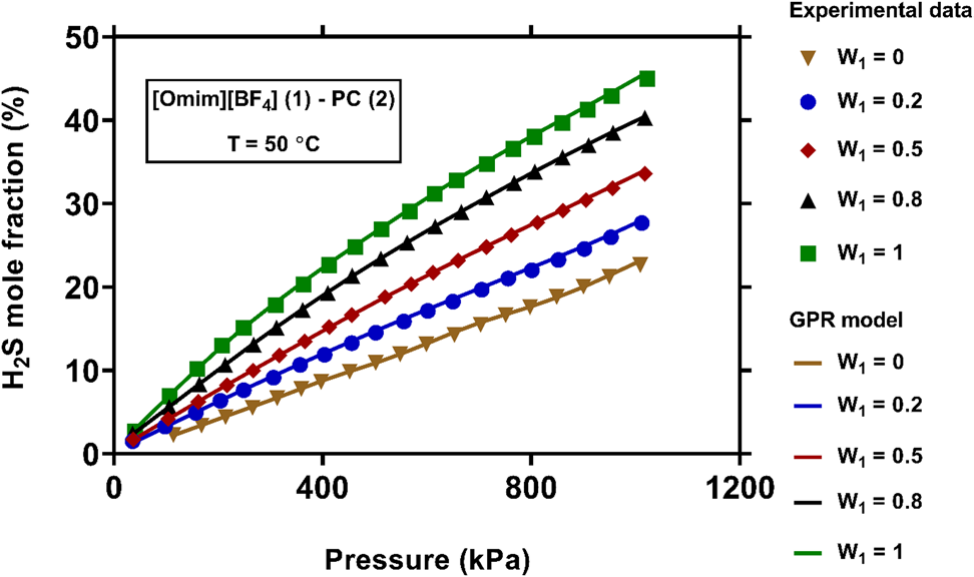
Comparison between experimental values of H_2_S solubility and those predicted by the novel model at various mass fractions of solvent components (created by GraphPad 8.4.3.686^145^).

Figure 9 illustrates the values of H_2_S solubility in the mixed solvents of [Omim][BF_4_]-PC at various mass fractions. It is seen that, existence of [Omim][BF_4_] ionic liquid in the solvent can significantly enhance the solubility of H_2_S. In addition, the pure [Omim][BF_4_] performs much better than pure PC in absorption of H_2_S gas. This fact is more obvious at the higher pressures. As can be seen, the GPR model precisely predicts the H_2_S solubility in the solvents with different mass fraction of components, and its outcomes are in close agreements with actual values.

**Figure 10:**
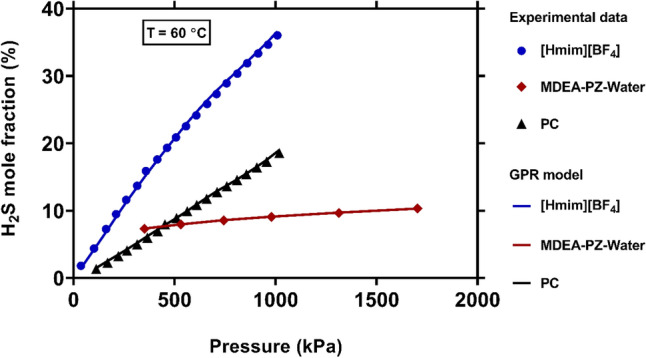
Comparison between experimental values of H_2_S solubility and those predicted by the novel model when using various types of solvents (created by GraphPad 8.4.3.686^145^).

A comparison between the solubility values of H_2_S in various types of solvents, including an ionic liquid ([Hmim][BF_4_]), an amine solution (MDEA-PZ-Water) and an organic solution (PC) is provided in Fig. 10. It is clear that under a same operating condition, the solubility of H_2_S in [Hmim][BF_4_] is much higher than that in other aforementioned solvents. Furthermore, the value of solubility in PC as solvent is higher than MDEA-PZ-Water ternary system. On the other hand, this figure reveals that while the solubility values in [Hmim][BF_4_] and PC strongly depend on pressure, there is no obvious relationship between pressure and solubility in MDEA-PZ-Water system. As seen, all these physical trends are excellently described by the novel model.

### Comparison with earlier machine learning based models

As discussed previously, the earlier machine learning based models for H_2_S solubility have been developed based on data for limited conditions and absorbers. A comparison between the performances and applicability ranges of the earlier models and the one established in this study is presented in Table 4. As seen, most of previous models have been suggested for single-component ionic liquids. Only that developed by Hamzehie et al.^71^ includes some data for binary and ternary mixtures of amines. In contrast, the databank analyzed in the current study covers enough experimental data for various types of solvents, such as amines, ionic liquids, electrolytes, organics, etc. Moreover, unlike the earlier studies, the data for single-component as well as binary, ternary and quaternary mixtures of solvents have been analyzed. On the other hand, while the number of data used for development of the novel model is extremely higher than the previous ones, its AARE of 4.73% for test data is still much reasonable. All these points reflect the fact that the model proposed by the novel approach of GPR can outperform all earlier ones in terms of universality, applicability and reliability.

**Table 4:** Comparison between the analyzed conditions as well as the performances of the previous models and the one developed by GPR approach.

Model	Machine learning method	Number of analyzed data	Number of solvents	Types of solvents	Number of components in analyzed solvents	Performance of the model
^72^	PSO-ANN	465	11	Ionic liquids	Single	AARE of 4.58% for all data
^76^	GP	465	11	Ionic liquids	Single	AARE of 3.93% for test data
^73^	GA-LSSVM	465	11	Ionic liquids	Single	AARE of 2.3% for all data
^71^	MLP	513	27	Amines, ionic liquids	Single, binary and ternary	AARE of 3.10% for test data
^46^	MLP	496	12	Ionic liquids	Single	AARE of 1.9% for all data
^37^	ELM	1134	27	Ionic liquids	Single	AARE of 5.78% for test data
^77^	MLP	664	13	Ionic liquids	Single	AARE of 11.68% for test data
^78^	CSA-LSSVM	664	14	Ionic liquids	Single	AARE of 0.15% for test data
^35^	SGB	465	11	Ionic liquids	Single	AARE of 7.54% for test data
^79^	ELM	1318	28	Ionic liquids	Single	AARE of 5.07% for test data
^36^	LSSVM	1298	27	Ionic liquids	Single	AARE of 2.74% for test data
^74^	MLP	1243	33	Ionic liquids	Single	AARE of 3.51% for test data
^80^	CMIS-GMDH	1243	33	Ionic liquids	Single	AARE of 2.77% for test data
^81^	CNN	1516	37	Ionic liquids	Single	AARE of 3.21% for test data
This study	GPR	5148	95	Amines, Ionic liquids, electrolytes, organics, etc.	Single, binary, ternary and quaternary	AARE of 4.73% for test data

### Sensitivity analysis

To clarify how each input factor affects the predictions of the novel model, a sensitivity analysis is performed in this section. Accordingly, the Pearson's correlation coefficients between the H_2_S solubility values calculated by GPR model and the input variables, i.e., temperature, pressure and equivalent molecular weight were calculated. It should be noted that the value of this index between two given variables, i.e., *X* and *Y* can be calculated using the following equation,

11 $$R\left(X,Y\right)=\frac{\sum_{i=1}^{n}\left({X}_{i}-\overline{{X}_{i}}\right)\left({Y}_{i}-\overline{{Y}_{i}}\right)}{\sqrt{\sum_{i=1}^{n}{\left({X}_{i}-\overline{{X}_{i}}\right)}^{2}\sum_{i=1}^{n}{\left({Y}_{i}-\overline{{Y}_{i}}\right)}^{2}}}$$

where n is the number of analyzed samples. In addition, $\overline{{X}_{i}}$ and $\overline{{Y}_{i}}$ are the averages values of *X* and *Y*, respectively. The values of − 1 and + 1 for this coefficient show the maximum levels of reverse and direct relationships between the corresponding variables, respectively. In contrast, when this value tends to zero, it can be found that there is no remarkable correlation between the variables.

**Figure 11:**
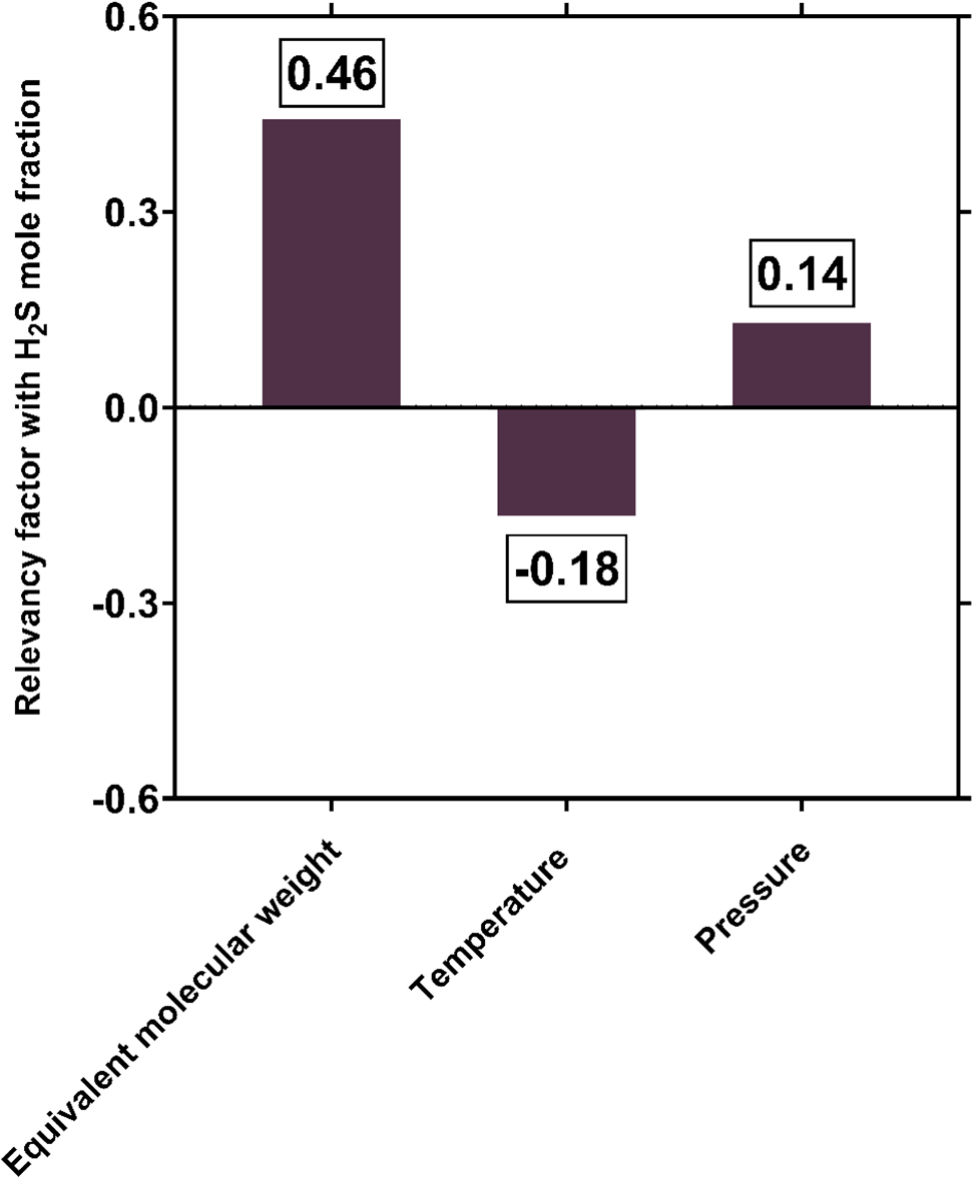
Relevancy factors between different input variables and the outcomes of GPR model for H_2_S solubility (created by GraphPad 8.4.3.686^145^).

Figure 11 depicts the Pearson's correlation coefficients between the input parameters and the outcomes of the GPR model. As observed, the operating pressure and equivalent molecular weight of solvent directly affect the H_2_S solubility. While, there is an inverse relationship between H_2_S solubility and temperature. The same results were also observed in section “Physical trends of H_2_S solubility”, based on experimental data and the outcomes of GPR model. On the other hand, Fig. 11 reveals that the equivalent molecular weight of solvent, which simultaneously involves both components' molecular weight and their mass fractions, is the most effective factor in controlling of H_2_S solubility. Moreover, operating temperature and pressure rank second and third, respectively in term of importance. Based on Fan et al.^42^ study, molecular weight has a significant impact on the ionic conductivity and the mechanism of ion transport. This fact can justify the great influence of equivalent molecular weight on solubility as observed here.

**Figure 12:**
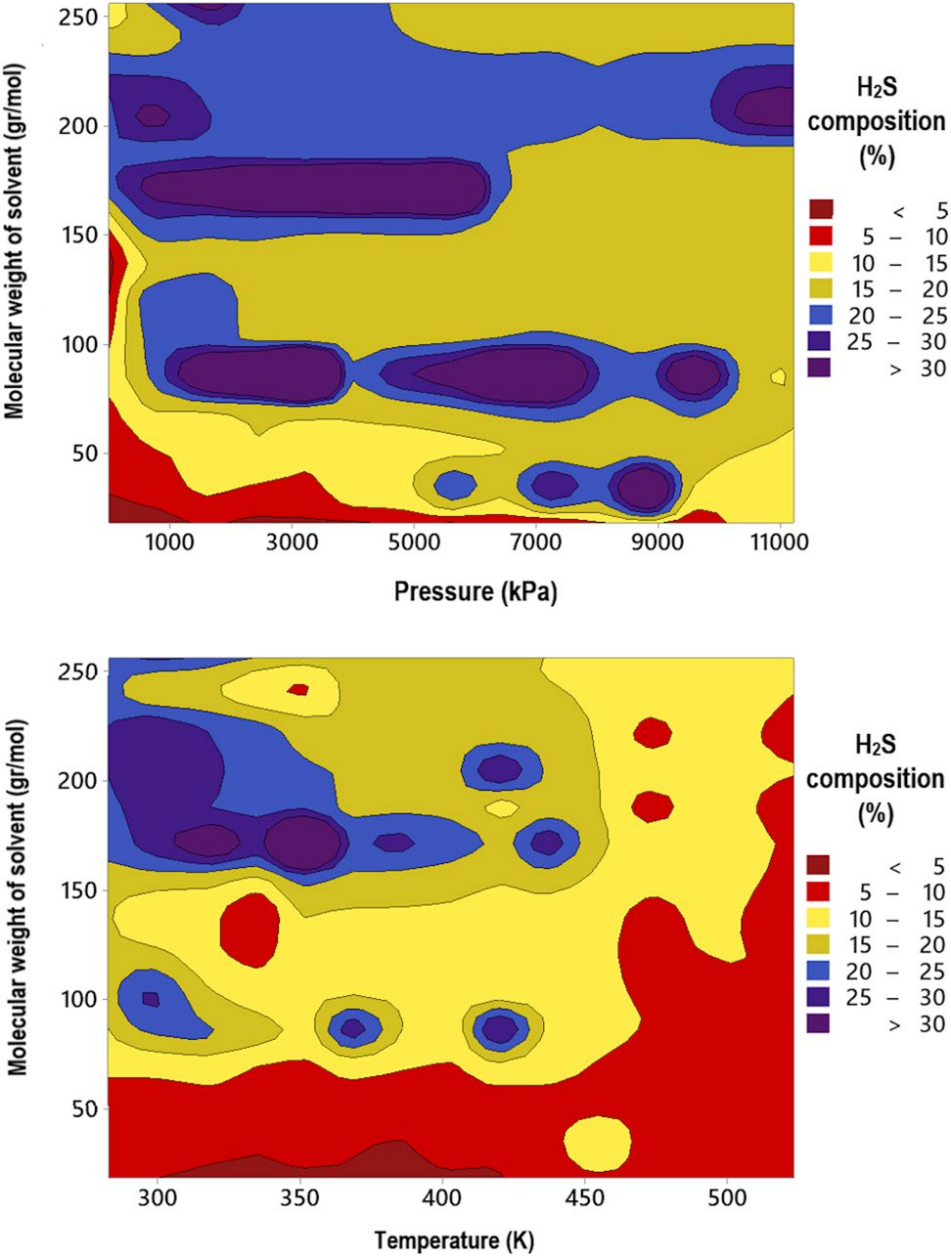
Contour plot of the alternation of H_2_S solubility over various ranges of pressures, temperatures and the molecular weights of solvents (created by Minitab 19^150^).

A contour plot of the correlation between the input variables and H_2_S solubility is shown in Fig. 12. Dark red to purple spectral colors indicate solubility changes from less than 5% to greater than 30%. The red and dark red areas are most commonly observed at high temperatures and low pressures when the molecular weight of the solvent is less than 100 g/mol. This means that the composition of H_2_S in the solvent is often less than 10% under these operating conditions. As the pressure increases and the temperature decreases, the solubility of H_2_S gradually increases and the yellow or blue areas become more prominent. The majority of the dark blue and purple colors are distributed in the area with high values for the molecular weight of solvent, which further exhibits that the molecular weight of the solvent has a much greater impact on controlling solubility than the other two factors. It should be mentioned that a solvent with a higher molecular weight can increase the efficiency of H_2_S abruption even in low pressure and high temperature environments. Overall, the findings of the current analysis are consistent with those of the prior sections.

## Conclusions

In the current study, robust approaches for predicting H_2_S solubility in solvents were developed based on machine learning algorithms of MLP, GPR and RBF. 5148 data points from 54 publications, enveloping broad pressure and temperature ranges, were used to establish and validate the novel models, which is the biggest databank analyzed to date. The analyzed databank covered 95 single and multicomponent solvents such as amines, ionic liquids, electrolytes, organics, etc. The main findings of this study are listed as:

- Among all machine learning based predictive approaches, GPR based model outperformed the others in predicting H_2_S solubility with AARE, ${\mathrm{R}}^{2}$ and RRMSE values of 4.73%, 99.75% and 4.83%, respectively, during the test process. This model predicted more than 87% of data in $\pm$ 10% error bounds. In contrast, the MLP and RBF models exhibited extremely larger deviations for the tested data samples with AAREs of 19.66% and 28.41%, and ${R}^{2}$ values of 94.61% and 90.91%, respectively.
- Based on the William's plot for GPR, it was determined that the experimental data analyzed in this study are highly reliable for developing new models, as the valid, good high leverage and doubtful data points covered 92.87%, 5.09% and 2.04% of the entire databank.
- The novel GPR model predicted the solubility data in organic solvents, ionic liquids, electrolytes, and amines with excellent AAREs of 2.49%, 2.87%, 5.19% and 6.91%, respectively. For other types of solvents, a total AARE of 5.73% was obtained by this approach. The GPR model also provided appropriate results for single-component, binary, ternary, and quaternary mixtures of solvents with AARE values of 2.88%, 5.39%, 6.45% and 4.69%, respectively. Moreover, this model was properly able to describe the physical trends of H_2_S solubility under various operating conditions.
- A comparison of the previous and the novel models implied that the GPR-based model was the preferable choice for estimating H_2_S solubility in terms of universality, applicability, and reliability.
- A sensitivity analysis based on the GPR model showed that equivalent molecular weight and operating pressure directly affect the H_2_S solubility, whereas the solubility is inversely related to temperature. Moreover, the equivalent molecular weight of solvent was known as the most influential factor with a relevancy factor of 0.46 with H_2_S solubility. Moreover, temperature and pressure ranked in second and third with relevance factors of − 0.18 and 0.14, respectively, with respect to H_2_S mole fraction. These results were also in line with the findings of the contour plot analysis.

## Data Availability

The datasets used and/or analyzed during the current study are available from the corresponding author on reasonable request.

## List of symbols

AARE: Average absolute relative error
Mw: Molecular weight of solvent, gr mol^−1^
P: Pressure, kPa
${R}^{2}$: Coefficient of determination
*R*_*i*_: Relative error of *i*th data from the actual value
RRMSE: Relative root mean squared error
T: Temperature, $$^\circ{\rm C} $$
${W}_{i}$: Mass fraction of *i*th component in the solvent
$${x}_{{H}_{2}S}$$: H_2_S mole fraction

## Subscripts

H_2_S: Related to H_2_S gas
eq: Equivalent
